# Association of the triglyceride–glucose index with coronary artery disease complexity in patients with acute coronary syndrome

**DOI:** 10.1186/s12933-023-01780-0

**Published:** 2023-03-12

**Authors:** Shiqiang Xiong, Qiang Chen, Yu Long, Hong Su, Yan Luo, Hanxiong Liu, Yingzhong Chen, Qiao Feng, Xiufen Peng, Maoling Jiang, Xiuqiong Yu, Zhen Zhang, Lin Cai

**Affiliations:** 1 Department of Cardiology, The Third People’s Hospital of Chengdu, Affiliated Hospital of Southwest Jiaotong University, Chengdu Cardiovascular Disease Research Institute, Chengdu, 610014 Sichuan China; 2 Department of Cardiology, Sichuan Mianyang 404 Hospital, Mianyang, 621000 Sichuan China

**Keywords:** Acute coronary syndrome, Coronary angiography, Diabetes mellitus, Prediabetes, Insulin resistance, Blood glucose, Triglyceride

## Abstract

**Aim:**

The triglyceride–glucose (TyG) index has been shown to be an independent predictor for the progression and prognosis of coronary artery disease (CAD). Whether the TyG index predicts the severity of CAD in patients presenting with acute coronary syndrome (ACS) remains unknown.

**Methods:**

A total of 1,007 individuals presenting with ACS undergoing coronary angiography were stratified according to the tertiles of the TyG index and The Synergy Between Percutaneous Coronary Intervention (SYNTAX) score (SYNTAX score ≤ 22 versus SYNTAX score > 22). CAD complexity was determined by the SYNTAX score.

**Results:**

After adjusting for multiple confounding factors, the TyG index was still an independent risk factor for mid/high SYNTAX scores (SYNTAX score > 22, OR 2.6452, 95% CI 1.9020–3.6786, P < 0.0001). Compared with the lowest tertile of the TyG (T1) group, the risk for a mid/high SYNTAX score in the T2 and T3 groups was 2.574-fold higher (OR, 2.574; 95% CI 1.610–4.112; P < 0.001) and 3.732-fold higher (OR, 3.732; 95% CI 2.330–5.975; P < 0.001), respectively. Furthermore, there was a dose‒response relationship between the TyG index and the risk of complicated CAD (SYNTAX score > 22; nonlinear P = 0.200). The risk for a mid/high SYNTAX score in the T2 and T3 groups was significantly higher in normoglycemia, prediabetes mellitus, and diabetes mellitus subgroups.

**Conclusions:**

A higher TyG index was associated with the presence of a higher coronary anatomical complexity (SYNTAX score > 22) in ACS patients, irrespective of diabetes mellitus status. The TyG index might serve as a noninvasive predictor of CAD complexity in ACS patients and could potentially influence the management and therapeutic approach.

**Supplementary Information:**

The online version contains supplementary material available at 10.1186/s12933-023-01780-0.

## Introduction

The Synergy Between Percutaneous Coronary Intervention (SYNTAX) score, a comprehensive angiographic tool that takes into account anatomic risk factors, is the best-known scoring system to grade the complexity of coronary artery disease (CAD) and objectively guide decision-making between coronary artery bypass grafting surgery and percutaneous coronary intervention (PCI) in patients with complex CAD [1]. According to the SYNTAX score, CAD patients are categorized as low (≤ 22), intermediate (23 to 32), or high risk (≥ 33) [2]. Patients with higher SYNTAX scores reflect more complex disease and are at higher potential risk of major adverse cardiovascular events [2, 3]. However, the calculation of the SYNTAX score depends on the findings of invasive coronary angiography. Noninvasively assessing the severity of CAD prior to coronary angiography could be beneficial for early stratification and possibly alter the therapeutic approach and management of patients with acute coronary syndrome (ACS).

Mounting evidence demonstrates that insulin resistance plays a crucial role in the development and pathogenesis of cardiovascular disease [4]. A high level of insulin resistance not only is associated with an increased risk of atherosclerotic cardiovascular disease but is also correlated with a higher risk of adverse cardiovascular events [5]. Recently, the triglyceride–glucose (TyG) index, derived from triglyceride and fasting blood glucose, has been shown to be a reliable surrogate marker of insulin resistance [6]. A recent large-scale prospective study suggested that the TyG index is an independent predictor for the progression of coronary artery calcification, especially in individuals without heavy coronary artery calcification at baseline [7]. Moreover, an increased TyG index has been shown to be independently associated with higher risks of atherosclerotic cardiovascular diseases, including myocardial infarction [8], and worse prognosis in patients with ACS, irrespective of diabetes mellitus [9].

Thus, the aim of this study was to investigate the angiographic severity of CAD encountered across the TyG index continuum and determine the association between the TyG index and the SYNTAX score in patients with ACS.

## Methods

### Study population

We enrolled 1,007 patients hospitalized at the Third People's Hospital of Chengdu (Sichuan, China) undergoing coronary angiography who were diagnosed with ACS from July 2018 to December 2020. Individuals with incomplete key variables, including the SYNTAX score and the TyG index variables, were excluded. This retrospective observational cohort study was approved by the local ethics committee and strictly adhered to the Declaration of Helsinki, with informed consent waived due to its retrospective nature.

### Data collection and definitions

Data on sociodemographic characteristics, medical history, smoking status, laboratory examination, and medical and procedural information of participants were collected from the electronic medical records. Previous medical history data included a history of PCI, hypertension, diabetes mellitus, atrial fibrillation, stroke, and chronic obstructive pulmonary disease. ACS was defined as including unstable angina (UA), non-ST segment elevation myocardial infarction (NSTEMI), or ST segment elevation myocardial infarction (STEMI) [10].

Peripheral venous blood samples from patients were collected after overnight fasting (> 8 h). Laboratory parameters, including fasting blood glucose (FBG), triglycerides (TG), total cholesterol (TC), low-density lipoprotein-C (LDL-C), high-density lipoprotein-C (HDL-C), cardiac troponin T (cTnT), serum creatinine (Scr), and brain natriuretic peptide (BNP), were measured by standard biochemical techniques in the Clinical Laboratory of the Third People's Hospital of Chengdu. The left ventricular ejection fraction (LVEF) was determined by the two-dimensional modified Simpson’s method.

The TyG index was calculated as ln [fasting TG (mg/dL) × FBG (mg/dL)/2] [11]. A web-based online calculation tool (http://syntaxscore.com/) was used to calculate the SYNTAX score from the preprocedural angiograms by two independent cardiologists who were blinded to the study protocol and baseline clinical characteristics.

### Statistical analysis

Categorical data are described as counts and percentages (%) and were compared via the chi-square or Fisher’s exact test when appropriate. Continuous data are described as the mean with standard deviation or median with interquartile range and were compared via Student’s t test or the Mann–Whitney U test, respectively. For comparisons across the tertiles of the TyG index, the one-way analysis of variance and the Kruskal–Wallis test for parametric and nonparametric variables were used for continuous variables, respectively, and the chi-square test was performed for categorical data.

Spearman’s correlation analysis was used to investigate the correlation between the SYNTAX score and other parameters. A logistic regression analysis was adopted to analyze the association between the TyG index and the angiographic severity of CAD (SYNTAX score ≤ 22 versus SYNTAX score > 22). After checking for collinearity, the variables with an unadjusted P value of < 0.05 were included in the multivariate model. The results are described as odds ratios (ORs) with 95% confidence intervals (CIs). Model I was adjusted for age, body mass index (BMI), hypertension, and diabetes mellitus. Model II was adjusted for age, BMI, hypertension, diabetes mellitus, heart rate (HR), BNP and Scr. Restricted cubic splines (RCS) were performed to evaluate the dose‒response relationship between the baseline TyG index and CAD severity.

The area under the receiver operating characteristic (ROC) curve (AUROC) were calculated to determine the diagnostic performance of the TyG index in detecting the severity of CAD in patients with ACS.

All statistical analyses were carried out with R version 4.0.2 software (R Foundation for Statistical Computing, Vienna, Austria) and SPSS version 26.0 software (IBM Corporation, New York, NY, USA). A P value < 0.05 was considered statistically significant.

## Results

### Baseline characteristics

The average age of the 1,007 patients (28.2% were female) with confirmed ACS (UA, NSTEMI, and STEMI) who underwent PCI was 66.55 ± 11.41 years. The baseline characteristics based on tertiles of the TyG index (T1, TyG ≤ 8.67; T2, 8.67 < TyG ≤ 9.18; T3, TyG > 9.18) are shown in Table 1. Compared with patients in the T1 group, patients with a higher TyG index tended to have a higher prevalence of diabetes mellitus, multivessel disease (MVD), left main lesion, and chronic total occlusion, increased levels of FBG, TG, TC, LDL-C, the TyG index and SYNTAX score, and a larger number and length of stents.

**Table 1:** The baseline characteristics based on tertiles of the TyG index

Variable	T1 (n = 341)	T2 (n = 331)	T3 (n = 335)	P value
Age, years	67.75 ± 11.64	65.80 ± 11.40	66.07 ± 11.13	0.055
Female, n (%)	72 (21.1)	87 (26.3)	125 (37.3)	< 0.001
BMI, kg/m^2^	23.63 ± 2.88	24.69 ± 2.73	24.64 ± 2.83	< 0.001
Smoking, n (%)	189 (55.4)	196 (59.2)	168(50.1)	0.061
Previous PCI, n (%)	32 (9.4)	25 (7.6)	27 (8.1)	0.674
COPD, n (%)	22 (6.5)	19 (5.7)	14 (4.2)	0.414
Hypertension, n (%)	216 (63.3)	219 (66.2)	220 (65.7)	0.714
Diabetes mellitus, n (%)	51 (15.0)	118 (35.6)	191 (57.0)	< 0.001
AF, n (%)	22 (6.5)	20 (6.0)	24 (7.2)	0.839
Previous Stroke, n (%)	28 (8.2)	25 (7.6)	24 (7.2)	0.874
SBP, mmHg	131.28 ± 19.96	132.73 ± 22.32	132.70 ± 22.00	0.602
HR, bpm	76.21 ± 14.13	78.14 ± 15.33	78.84 ± 14.79	0.056
cTnT, pg/ml	32.53 (11.18, 899.45)	30.47 (11.50, 534.50)	64.79 (13.48, 1178.00)	0.094
BNP, pg/ml	109.90 (46.45, 300.85)	95.5 (31.6, 242.75)	118.75 (39.6, 379.85)	0.028
Scr, umol/L	77.60 (66.25, 90.30)	77.00 (65.60, 91.60)	73.90 (61.30, 93.10)	0.335
FBG, mmol/L	5.44 ± 1.20	6.46 ± 1.79	8.97 ± 3.58	< 0.001
TG, mmol/L	0.99 ± 0.29	1.61 ± 0.42	2.22 ± 0.89	< 0.001
TC, mmol/L	4.09 ± 1.09	4.59 ± 1.29	4.75 ± 1.19	< 0.001
HDL-C, mmol/L	1.22 ± 0.33	1.12 ± 0.26	1.12 ± 0.27	< 0.001
LDL-C, mmol/L	2.49 ± 0.82	2.87 ± 0.95	2.95 ± 0.86	< 0.001
AMI, n (%)	174 (51.0)	171 (51.7)	193 (57.6)	0.168
Diagnosis, n (%)				0.137
UA	167 (49.0)	160 (48.3)	142 (42.4)	
NSTEMI	65 (19.1)	81 (24.5)	83 (24.8)	
STEMI	109 (32.0)	90 (27.2)	110 (32.8)	
Angiographic data				
MVD, n (%)	204 (59.8)	228 (68.9)	253 (75.5)	< 0.001
LM, n (%)	4 (1.2)	23(6.9)	27 (8.1)	< 0.001
Calcified lesions, n (%)	41 (12.0)	41(12.4)	56 (16.7)	0.144
Thrombosis, n (%)	24 (7.0)	24(7.3)	36 (10.7)	0.149
Long lesion, n (%)	132 (38.7)	149(45.0)	177 (52.8)	0.001
CTO, n (%)	57 (16.7)	69(20.8)	82 (24.5)	0.045
Number of stents	1.32 ± 0.75	1.42 ± 0.88	1.63 ± 0.99	< 0.001
Length of stents, mm	34.12 ± 22.73	37.02 ± 26.50	43.94 ± 29.75	< 0.001
bSS	11.00 (7.00, 17.5)	13(8.00, 20.00)	16.00 (10.00, 23.50)	< 0.001
TyG index	8.30 ± 0.30	8.95 ± 1.15	9.54 ± 0.32	< 0.001

The groups were stratified by the tertiles of the TyG index (T1, TyG ≤ 8.67; T2, 8.67 < TyG ≤ 9.18; T3, TyG > 9.18). *BMI* body mass index, *COPD* chronic obstructive pulmonary disease, *AF* atrial fibrillation, *SBP* systolic blood pressure, *HR* heart rate, *BNP* brain natriuretic peptide, *Scr* serum creatinine, *FBG* fasting blood glucose, *TG* triglyceride, *TC* total cholesterol, *HDL-C* high density lipoprotein, *LDL-C* low density lipoprotein, *UA* unstable angina, *STEMI* ST-segment elevation myocardial infarction, *NSTEMI* non-ST-segment elevation myocardial infarction, *MVD* multivessel disease, *LM* left main disease, *CTO* chronic total occlusion, *bSS* baseline SYNTAX score, TyG index, the triglyceride–glucose index. Data are presented as mean ± SD, median (IQR) or n (%)

According to the SYNTAX score, patients were categorized into low (SYNTAX score ≤ 22) and mid/high risk (SYNTAX score > 22) groups as shown in Additional file 1: Table S1. Patients with a SYNTAX score > 22 were older and had higher prevalence rates of hypertension, diabetes mellitus, AMI, MVD, left main lesion, calcified lesions, thrombosis, long lesion, and chronic total occlusion, increased levels of HR, cTnT, BNP, Scr, FBG, TG, and the TyG index, and a larger number and length of stents.

### Association between the TyG index and severity of CAD

The univariate logistic regression analysis revealed that age, BMI, previous history of hypertension and diabetes mellitus, HR, BNP, Scr, FBG, TGs, and the TyG index ﻿were potential risk factors (univariate P < 0.05) for a mid/high SYNTAX score (SYNTAX score > 22, Table 2). FBG and TG, being components of the TyG index, were not included in the multivariable logistic regression model in order to avoid any potential interactions. After checking for collinearity, the potential risk factors were used as variables in the multivariate model, and the results showed that the TyG index was an independent predictor of the mid/high SYNTAX score (SYNTAX score > 22, OR 2.6452, 95% CI 1.9020–3.6786, P < 0.0001).

**Table 2:** Univariate and multivariate logistic regression analysis for predicting a mid/high SYNTAX score

Variables	Univariate analysis			Multivariate analysis		
	OR	95% CI	P value	OR	95% CI	P value
Age, years	1.0366	1.0211–1.0523	< 0.0001	1.0303	1.0135–1.0474	0.0004
Female, n (%)	1.0937	0.7743–1.5449	0.6113			
BMI, kg/m^2^	0.9297	0.8788–0.9836	0.0113	0.9334	0.8772–0.9932	0.0295
Smoking, n (%)	0.8392	0.6100–1.1545	0.2814			
Previous PCI, n (%)	0.9162	0.5120–1.6396	0.7683			
Hypertension, n (%)	1.4282	1.0126–2.0143	0.0422	1.3437	0.9187–1.9653	0.1277
Diabetes mellitus, n (%)	1.4790	1.0737–2.0371	0.0166	0.8930	0.6150–1.2966	0.5520
AF, n (%)	1.6530	0.9384–2.9118	0.0819			
SBP, mmHg	0.9966	0.9892–1.0040	0.3675			
HR, bpm	1.0108	1.0005–1.0212	0.0404	1.0049	0.9941–1.0158	0.3714
cTnT, pg/ml	1.0000	1.0000–1.0001	0.0863			
BNP, pg/ml	1.0005	1.0003–1.0007	< 0.0001	1.0004	1.0002–1.0006	0.0009
Scr, umol/L	1.0021	1.0007–1.0034	0.0031	1.0013	0.9999–1.0026	0.0677
TyG index	2.4187	1.8081–3.2357	< 0.0001	2.6452	1.9020–3.6786	< 0.0001
FBG, mmol/L	1.0911	1.0377–1.1471	0.0007			
TG, mmol/L	1.7999	1.4548–2.2270	< 0.0001			
TC, mmol/L	1.1062	0.9758–1.2539	0.1148			
HDL-C, mmol/L	1.0840	0.6414–1.8322	0.7632			
LDL-C, mmol/L	1.0684	0.9847–1.1593	0.1119			

Abbreviations as shown in Table 1. *PCI* percutaneous coronary intervention, Data are presented as mean ± SD, median (IQR) or n (%)

Logistic regression models were further constructed to demonstrate that the TyG index was significantly associated with CAD severity (P < 0.001, Table 3). When analyzed as a continuous variable, the TyG index was significantly related to a mid/high score (SYNTAX score > 22, OR: 2.419; 95% CI 1.808–3.236, P < 0.001). Using the T1 group as a reference, the risk of a mid/high SYNTAX score for the T2 and T3 groups was 2.110-fold higher (OR, 2.110; 95% CI 1.361–3.270; P < 0.001) and 3.112-fold higher (OR, 3.112; 95% CI 2.042–4.744; P < 0.001), respectively. After adjusting for age, BMI, hypertension, diabetes mellitus, HR, BNP and Scr, the TyG index as a categorical variable was still an independent hazard factor for a mid/high SYNTAX score (OR, 2.645; 95% CI 1.902–3.679; P < 0.001). Compared with the T1 group, the risk for a mid/high SYNTAX score in the T2 and T3 groups was 2.574-fold higher (OR, 2.574; 95% CI 1.610–4.112; P < 0.001) and 3.732-fold higher (OR, 3.732; 95% CI 2.330–5.975; P < 0.001), respectively.

**Table 3:** Associations between the TyG index and complexity of CAD

	Non-adjusted		Model I		Model II	
	OR (95% CI)	P value	OR (95% CI)	P value	OR (95% CI)	P value
TyG index	2.419 (1.808–3.236)	< 0.001	2.719(1.965–3.762)	< 0.001	2.645 (1.902–3.679)	< 0.001
T1	Ref.	Ref.	Ref.	Ref.	Ref.	Ref.
T2	2.110 (1.361–3.270)	< 0.001	2.480(1.571–3.917)	< 0.001	2.574 (1.610–4.112)	< 0.001
T3	3.112 (2.042–4.744)	< 0.001	3.671(2.320–5.809)	< 0.001	3.732 (2.330–5.975)	< 0.001

None, non-adjusted model. Model I was adjusted for age, BMI, hypertension, diabetes mellitus, Model II was adjusted for age, BMI, hypertension, diabetes mellitus, heart rate, BNP and serum creatinine. *CAD* coronary artery disease

**Fig. 1:**
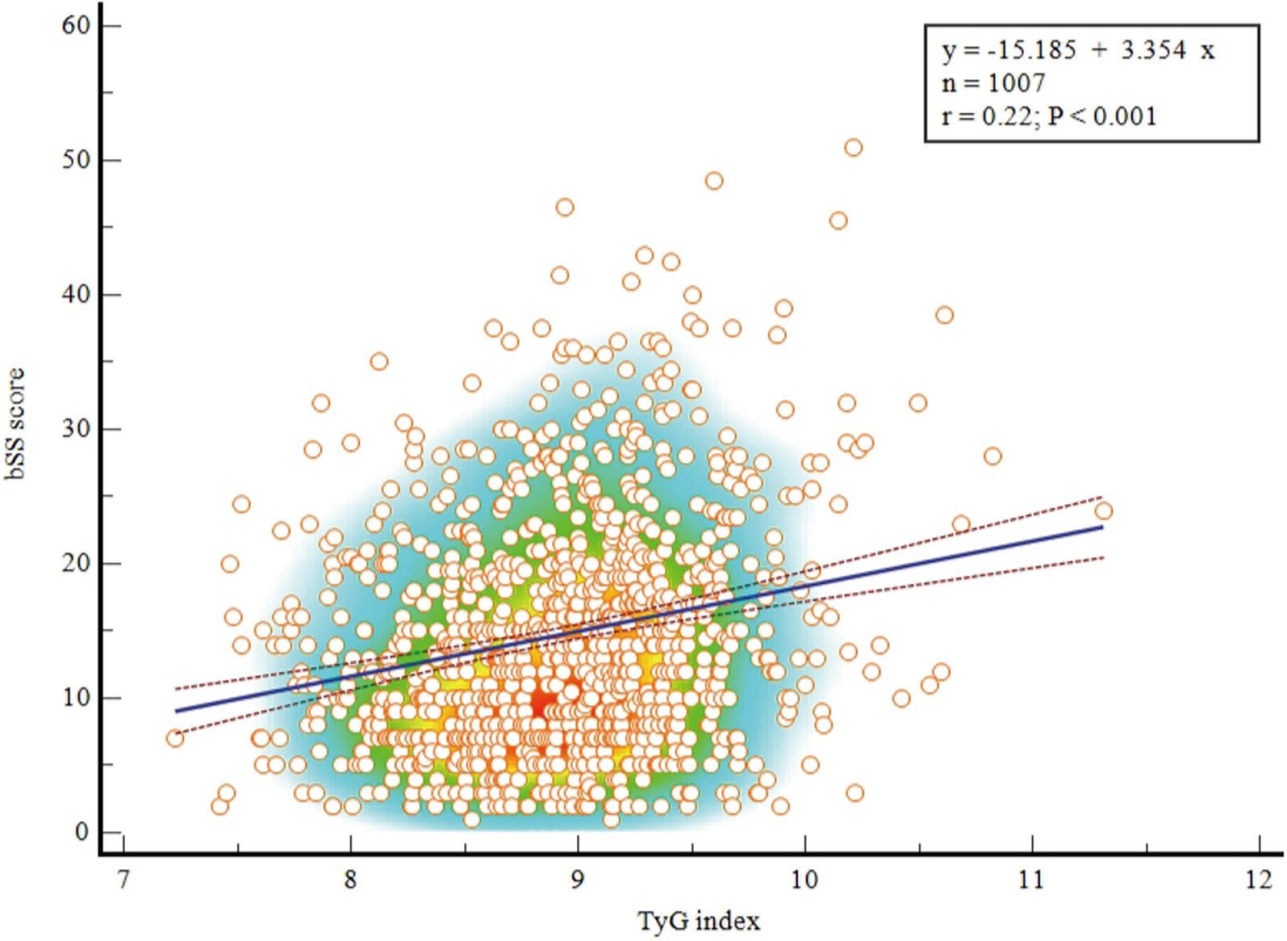
Correlation of the SYNTAX score with the TyG index. Spearman’s correlation analysis found that there was a significant positive correlation between the TyG index and the SYNTAX scores (r = 0.22, P < 0.001). *SYNTAX score* Synergy Between Percutaneous Coronary Intervention score, *TyG index* triglyceride–glucose index

**Fig. 2:**
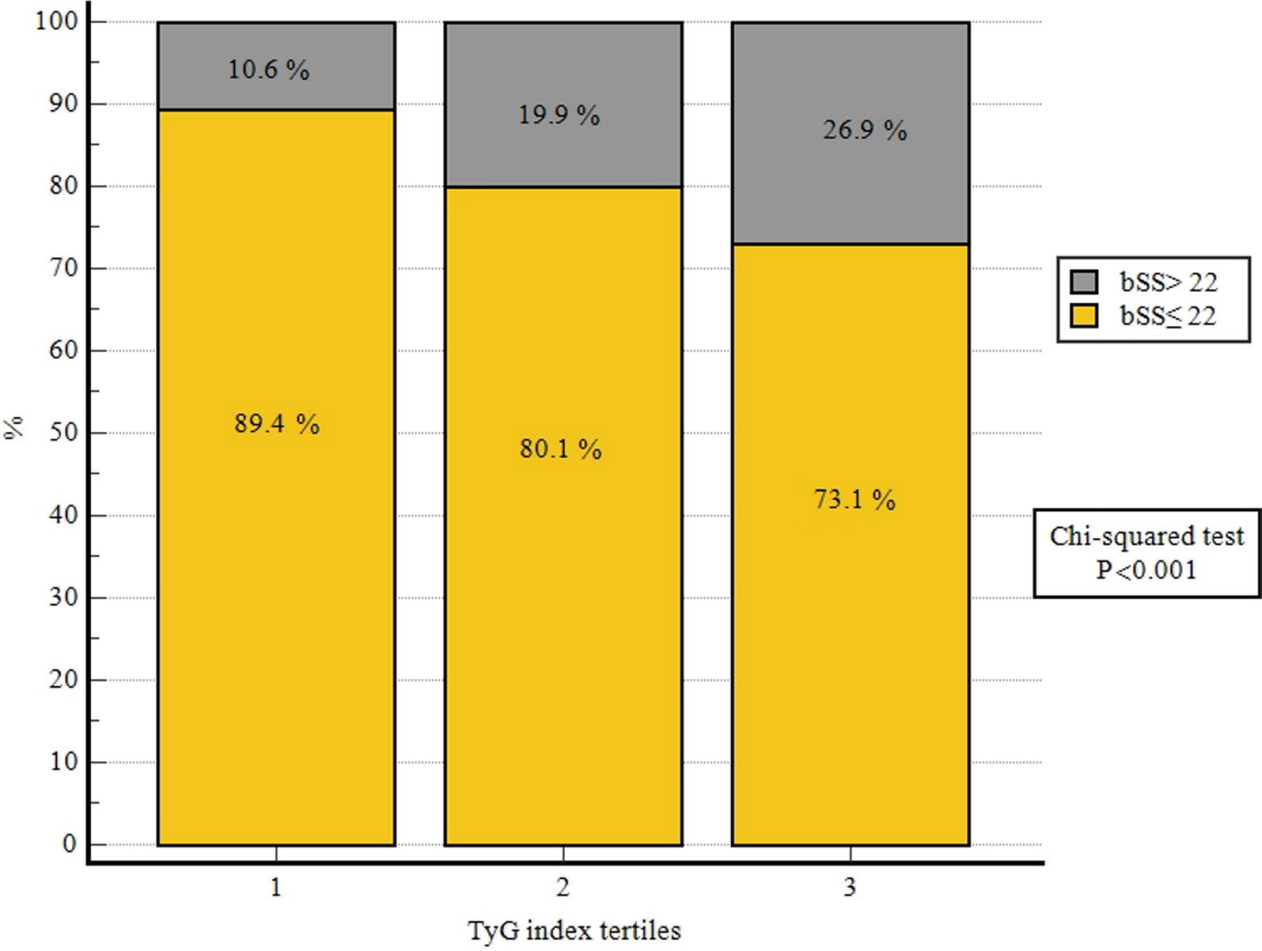
Comparison of the SYNTAX score according to the TyG index tertiles. The proportion of patients with a SYNTAX score ≤ 22 and SYNTAX score > 22 in patients presenting with acute coronary syndrome stratified according to the tertiles of the TyG index. *SYNTAX score* Synergy Between Percutaneous Coronary Intervention score, *TyG index* triglyceride–glucose index

**Fig. 3:**
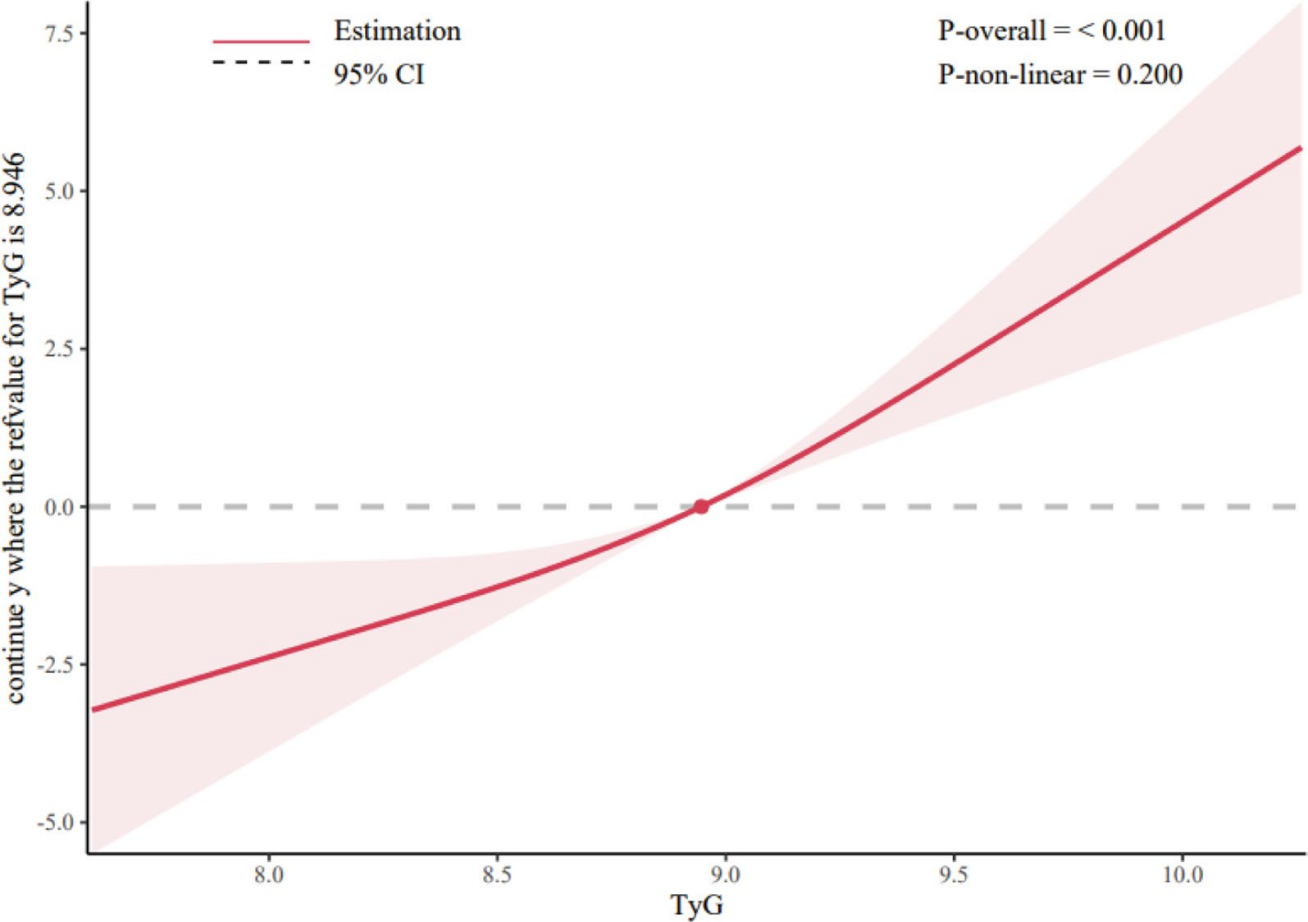
RCS for the odds ratio of a mid/high SYNTAX score. *RCS* restricted cubic spline, *OR* odds ratio, *SYNTAX score* Synergy Between Percutaneous Coronary Intervention score

The spearman’s correlation analysis found that there was a statistically significant but weak positive correlation between the TyG index and the SYNTAX scores (r = 0.22, P < 0.001, Fig. 1). Compared with patients in the T1 group, the proportion of patients with a SYNTAX score > 22 was larger in participants with a higher TyG index (Fig. 2). Additionally, the results of the RCS showed a dose‒response relationship between the TyG index and the risk of a mid/high SYNTAX score (Fig. 3; Nonlinear P = 0.200).

### The predictive performance of the TyG index for complex coronary lesions

**Fig. 4:**
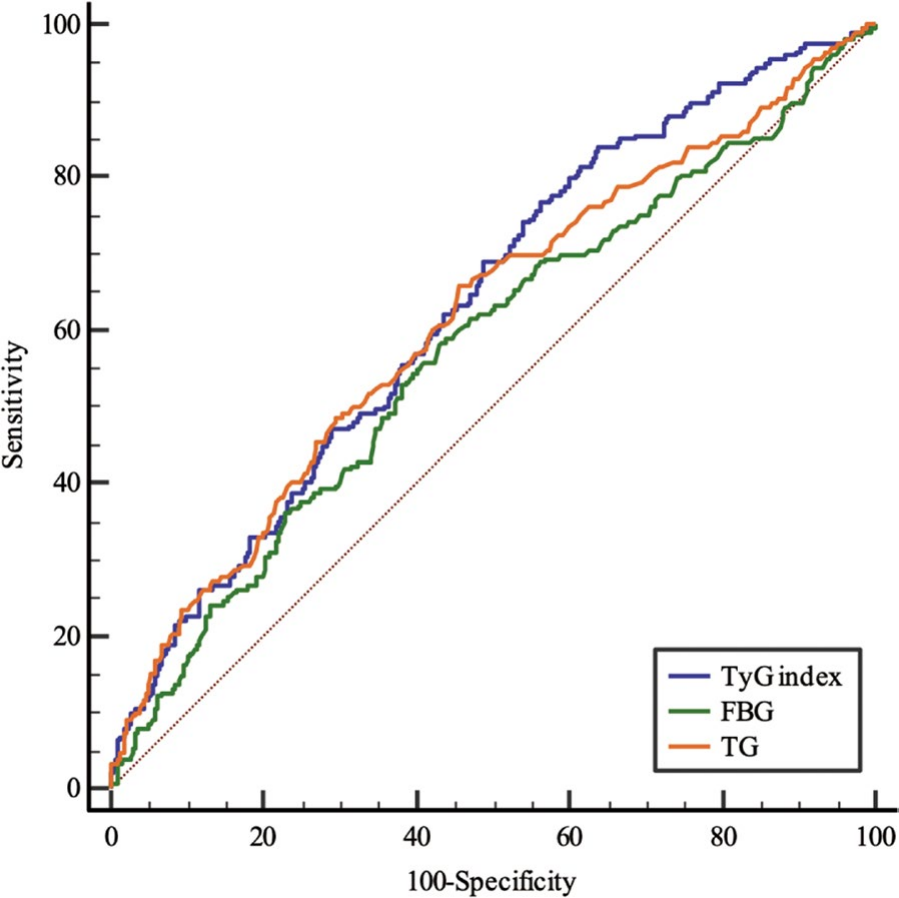
ROC curves for predicting a mid/high SYNTAX score. The area under the ROC curve of the TyG index, FBS, and TG for predicting a mid/high SYNTAX score (> 22) was 0.631 (95% CI 0.588–0.674, P < 0.001), 0.574 (95% CI 0.528–0.621, P = 0.002), and 0.613 (95% CI 0.567–0.659, P < 0.001), respectively. *SYNTAX score* Synergy Between Percutaneous Coronary Intervention score, *TyG index* triglyceride–glucose index, *ROC curve* receiver operating characteristic curve, *FBG* fasting blood glucose, *TG* triglyceride

The AUROC of the TyG index was significantly higher than that of fasting blood glucose (0.631 [95% CI 0.588–0.674] vs. 0.574 [95% CI 0.528–0.621], P = 0.0095) and was greater than that of TG with no statistical significance (0.631 [95% CI: 0.588–0.674] vs. 0.613 [95% CI 0.567–0.659], P = 0.2651) (Fig. 4) (Additional file 1: Table S2). These results demonstrated the TyG index has the highest predictive value for predicting coronary anatomical complexity (SYNTAX score > 22) in patients with ACS, when compared to either FBS or TG alone.

### Associations between the TyG index and the severity of CAD in subgroups stratified by different glucose metabolism status

Subgroup analyses were also conducted to investigate the associations between the TyG index and the severity of CAD in patients according to different diabetes status, including those with normoglycemia (n = 363), prediabetes mellitus (n = 284), and diabetes mellitus (n = 360) (Table 4). When adjusted for age, BMI, hypertension, heart rate, BNP, and serum creatinine in model II, the TyG index as a continuous variable was an independent risk factor for a mid/high SYNTAX score in individuals with normoglycemia (OR, 2.902; 95% CI 1.453–5.797; P = 0.003), prediabetes mellitus (OR, 2.321; 95% CI 1.213–4.441; P = 0.011), and diabetes mellitus (OR, 2.666; 95% CI 1.585–4.486; P < 0.001). Compared with the T1 group, the risk for a mid/high SYNTAX score in the T2 and T3 groups was significantly higher in all subgroups, irrespective of diabetes mellitus status.

**Table 4:** Associations between the TyG index and severity of CAD in different glucose metabolism status

	Non-adjusted		Model I		Model II	
	OR (95% CI)	P value	OR (95% CI)	P value	OR (95% CI)	P value
NG						
TyG index	2.159 (1.140–4.092)	0.018	2.843(1.442–5.606)	0.003	2.902(1.453–5.797)	0.003
T1	Ref.	Ref.	Ref.	Ref.	Ref.	Ref.
T2	2.140(1.088–4.208)	0.027	2.705(1.335–5.480)	0.006	2.654 (1.282–5.492)	0.009
T3	2.562(1.105–5.945)	0.028	3.221(1.334–7.779)	0.009	3.544(1.446–8.686)	0.006
Pre-DM						
TyG index	2.091(1.126–3.884)	0.020	2.095(1.123–3.910)	0.020	2.321(1.213–4.441)	0.011
T1	Ref.	Ref.	Ref.	Ref.	Ref.	Ref.
T2	1.412(0.642–3.103)	0.391	1.503(0.675–3.348)	0.319	1.789 (0.771–4.147)	0.176
T3	2.669(1.261–5.648)	0.010	2.784(1.300–5.964)	0.008	3.309(1.481–7.391)	0.004
DM						
TyG index	2.577 (1.591–4.175)	< 0.001	2.957 (1.782–4.907)	< 0.001	2.666 (1.585–4.486)	< 0.001
T1	Ref.	Ref.	Ref.	Ref.	Ref.	Ref.
T2	2.730 (0.986–0.755)	0.053	3.595(1.262–10.241)	0.017	3.143 (1.084–9.113)	0.035
T3	3.351 (1.262–8.903)	0.015	4.867(1.772–13.365)	0.002	4.029(1.445–11.231)	0.008

None, non-adjusted model. Model I was adjusted for age, BMI, hypertension, diabetes mellitus, Model II was adjusted for age, BMI, hypertension, diabetes mellitus, heart rate, BNP and serum creatinine. *CAD* coronary artery disease, *NG* normoglycemia, *Pre-DM* prediabetes mellitus, *DM* prediabetes mellitus

## Discussion

The present study shows that a higher TyG index independently predicts the presence of a higher coronary anatomical complexity (SYNTAX score > 22) in patients with ACS undergoing coronary angiography, irrespective of diabetes mellitus status. Our findings suggest that a higher insulin resistance represented by the TyG index makes the patients more susceptible to severe coronary lesions.

The adverse effect on the clinical prognosis of more extensive and complex CAD has been confirmed in many studies. Fumiaki et al. demonstrated that a mid/high SYNTAX score (≥ 23) could predict increased risks of major cardiovascular events (HR 1.36; 95% CI 1.07–1.75, P = 0.01) over 5 years in patients from the BARI-2D trial [3]. Additionally, higher SYNTAX scores were significantly associated with more favorable outcomes of revascularization compared with medical therapy among patients suitable for coronary artery bypass grafting surgery [3]. Higher SYNTAX scores also predicted a particular therapeutic benefit from coronary artery bypass grafting surgery compared with PCI in the SYNTAX trial [12]. The most recent clinical guideline for coronary artery revascularization recommends that using the SYNTAX score to assess CAD complexity in patients with multivessel CAD may be useful to guide revascularization [13]. Nevertheless, the application of the SYNTAX score in early treatment decisions in patients with ACS is less clear, because its calculation has to depend on the findings of invasive coronary angiography.

The key finding of this study is that higher levels of the TyG index predict more extensive and complex coronary anatomical lesions in patients with ACS, irrespective of diabetes mellitus status, and this index score can be determined in a non-invasive manner. Previously, although Wang et al. found a significant association between the TyG index and the incidence of MVD, the results were only significant in patients with prediabetes mellitus [14]. Additionally, Lee et al. reported that the TyG index was associated with an increased risk of coronary artery stenosis in asymptomatic subjects with type 2 diabetes mellitus, and the degree of coronary artery stenosis was not quantified [15]. Notably, in the present study, a significantly higher complexity of CAD, including MVD, left main lesion, calcified lesions, thrombosis, long lesion, and chronic total occlusion increased with increasing TyG index levels. Further analysis demonstrated that the associations between the TyG index and the severity of CAD were significant in both diabetic and nondiabetic individuals. These findings indicate that the TyG index might serve as a predictor of CAD severity in patients with ACS prior to undergoing coronary angiography.

Mounting epidemiological evidence suggests that insulin resistance constitutes an independent prognostic predictor in CAD [16], but insulin resistance or its surrogate marker has not been included in any risk prediction tools, such as the GRACE score or the SYNTAX score. Previous studies have shown that prediction models combining anatomical and clinical factors such as the SYNTAX II score and clinical residual SYNTAX score could improve the discriminative ability for a better risk assessment [17–19]. One of our previous studies also highlighted that adjustment of the residual SYNTAX score by the TyG index significantly improves the predictive accuracy for adverse cardiovascular events in patients with type 2 diabetes mellitus undergoing PCI [11]. Therefore, we suggest that the TyG index could be added to a preexisting risk prediction model to enhance its discriminate ability for patients with CAD in future studies.

Although the detailed mechanism underlying the association between the TyG index and cardiovascular disease is not fully illustrated, the TyG index has been regarded as a valuable indicator linked to insulin resistance and cardiovascular disease. Chronic hyperglycemia and dyslipidemia induced by insulin resistance contribute to the development of cardiovascular disease [5]. A higher level of TyG index has been shown to be associated with an increased risk of cardiovascular diseases in the general population [8, 20–22], and is an independent predictor of poor prognosis in different cohorts undergoing PCI [23–29]. One of our previous studies demonstrated that the TyG index could provide additional predictive ability on the top of residual SYNTAX score in predicting intermediate‑term major adverse cardiovascular events after PCI in patients with diabetes mellitus [11]. Recently, Wang et al. revealed that the TyG index was an independent risk factor for multi-vessel coronary artery disease in individuals with prediabetes mellitus, but not in those with normoglycemia or diabetes mellitus [14]. Meanwhile, another literature reported that the association between the TyG index and multi-vessel coronary artery disease was significant in patients with diabetes mellitus [30]. In line with previous studies, the current research supports this notion by more thoroughly demonstrating that the TyG index could independently predict a mid/high SYNTAX score (≥ 23) in patients with ACS, irrespective of diabetes mellitus status. Taken together, these findings indicated that a higher insulin resistance represented by the TyG index makes individuals more susceptible to severe coronary lesions and unfavorable outcomes.

Recently, several cardiovascular outcome trials have demonstrated that therapies aimed at improving insulin resistance are a promising intervention for diabetic patients at risk of experiencing adverse cardiovascular events [31]. Pioglitazone, a potent insulin sensitizer, has been shown to reduce atherosclerotic progression (based on PERISCOPE and Chicago studies) and the rate of cardiovascular events (according to the IRIS and PROactive randomized prospective cardiovascular outcome trials) [32–35]. Glucagon-like peptide-1 analogues have been shown to reduce the risk of major adverse cardiac events and have a direct impact on cardiac mortality in advanced atherosclerosis [36, 37]. The potential cardioprotective effect of glucagon-like peptide-1 analogues is partially attributed to their direct effects on vascular redox state and changes in insulin resistance [37]. Therefore, treatment of insulin resistance may contribute to the amelioration of coronary lesions and clinical prognosis.

## Limitations

This is a single-center, observational study with a relatively small sample size and that only enrolled the Chinese population. The results should be interpreted cautiously and further verified by multicenter and large sample size studies. Additionally, because of the inevitable inherent disadvantage of retrospective studies, a causal relationship between the TyG index and CAD complexity could not be concluded from this study; therefore, these findings need to be verified by a prospective study. Additionally, the feasibility of calculating TyG Index using blood samples collected after an overnight fasting (> 8 h) before coronary angiography is limited in some patients undergoing an emergent coronary angiography, particularly those with STEMI.

## Conclusions

The present study demonstrated a significantly positive relationship between the TyG index and the SYNTAX scores in patients with ACS undergoing coronary angiography. A higher TyG index independently predicted the presence of a higher coronary anatomical complexity (SYNTAX score > 22) in patients with ACS, irrespective of diabetes mellitus status. Our findings suggest that the TyG index could be used as a predictor of CAD severity and could potentially influence the management and therapeutic approach. Novel therapies aimed at improving insulin resistance may contribute to the amelioration of coronary lesions and clinical prognosis.

## Supplementary Information

**Additional file 1: Table S1.** The baseline characteristics based on tertiles of the baseline SYNTAX score.** Table S2.** Comparisons of the area under the ROC curves of the TyG index, FBG and TG.

## Data Availability

The datasets used and/or analyzed in the study are available from the corresponding author upon reasonable request.

## Abbreviations

CAD: Coronary artery disease
SYNTAX score: The Synergy Between Percutaneous Coronary Intervention score
BMI: Body mass index
COPD: Chronic obstructive pulmonary disease
AF: Atrial fibrillation
SBP: Systolic blood pressure
HR: Heart rate
BNP: Brain natriuretic peptide
Scr: Serum creatinine
FBG: Fasting blood glucose
TG: Triglyceride
TC: Total cholesterol
HDL-C: High density lipoprotein
LDL-C: Low density lipoprotein
UA: Unstable angina
STEMI: ST-segment elevation myocardial infarction
NSTEMI: Non-ST-segment elevation myocardial infarction
MVD: Multivessel disease
LM: Left main disease
CTO: Chronic total occlusion
bSS: Baseline SYNTAX score
TyG index: The triglyceride–glucose index

## References

[1] Sianos G, Morel MA, Kappetein AP, Morice MC, Colombo A, Dawkins K, van den Brand M, Van Dyck N, Russell ME, Mohr FW (2005). The SYNTAX Score: an angiographic tool grading the complexity of coronary artery disease. EuroIntervention.

[2] Serruys PW, Morice MC, Kappetein AP, Colombo A, Holmes DR, Mack MJ, Ståhle E, Feldman TE, van den Brand M, Bass EJ (2009). Percutaneous coronary intervention versus coronary-artery bypass grafting for severe coronary artery disease. N Engl J Med.

[3] Ikeno F, Brooks MM, Nakagawa K, Kim MK, Kaneda H, Mitsutake Y, Vlachos HA, Schwartz L, Frye RL, Kelsey SF (2017). SYNTAX Score and long-term outcomes: the BARI-2D Trial. J Am Coll Cardiol.

[4] Hill MA, Yang Y, Zhang L, Sun Z, Jia G, Parrish AR, Sowers JR (2021). Insulin resistance, cardiovascular stiffening and cardiovascular disease. Metabolism.

[5] Ormazabal V, Nair S, Elfeky O, Aguayo C, Salomon C, Zuñiga FA (2018). Association between insulin resistance and the development of cardiovascular disease. Cardiovasc Diabetol.

[6] Sanchez-Garcia A, Rodriguez-Gutierrez R, Mancillas-Adame L, Gonzalez-Nava V, Diaz Gonzalez-Colmenero A, Solis RC, Alvarez-Villalobos NA, Gonzalez-Gonzalez JG (2020). Diagnostic accuracy of the triglyceride and glucose index for insulin resistance: a systematic review. Int J Endocrinol.

[7] Won KB, Park EJ, Han D, Lee JH, Choi SY, Chun EJ, Park SH, Han HW, Sung J, Jung HO (2020). Triglyceride glucose index is an independent predictor for the progression of coronary artery calcification in the absence of heavy coronary artery calcification at baseline. Cardiovasc Diabetol.

[8] Tian X, Zuo Y, Chen S, Liu Q, Tao B, Wu S, Wang A (2021). Triglyceride-glucose index is associated with the risk of myocardial infarction: an 11-year prospective study in the Kailuan cohort. Cardiovasc Diabetol.

[9] Xiong S, Chen Q, Chen X, Hou J, Chen Y, Long Y, Yang S, Qi L, Su H, Huang W (2022). Adjustment of the GRACE score by the triglyceride glucose index improves the prediction of clinical outcomes in patients with acute coronary syndrome undergoing percutaneous coronary intervention. Cardiovasc Diabetol.

[10] Ohman EM, Granger CB, Harrington RA, Lee KL (2000). Risk stratification and therapeutic decision making in acute coronary syndromes. JAMA.

[11] Xiong S, Chen Q, Zhang Z, Chen Y, Hou J, Cui C, Cheng L, Su H, Long Y, Yang S (2022). A synergistic effect of the triglyceride-glucose index and the residual SYNTAX score on the prediction of intermediate-term major adverse cardiac events in patients with type 2 diabetes mellitus undergoing percutaneous coronary intervention. Cardiovasc Diabetol.

[12] Mohr FW, Morice MC, Kappetein AP, Feldman TE, Ståhle E, Colombo A, Mack MJ, Holmes DR, Morel MA, Van Dyck N (2013). Coronary artery bypass graft surgery versus percutaneous coronary intervention in patients with three-vessel disease and left main coronary disease: 5-year follow-up of the randomised, clinical SYNTAX trial. Lancet.

[13] Lawton JS, Tamis-Holland JE, Bangalore S, Bates ER, Beckie TM, Bischoff JM, Bittl JA, Cohen MG, DiMaio JM, Don CW (2022). 2021 ACC/AHA/SCAI Guideline for coronary artery revascularization: executive summary: a report of the american college of cardiology/american heart association joint committee on clinical practice guidelines. Circulation.

[14] Wang X, Xu W, Song Q, Zhao Z, Meng X, Xia C, Xie Y, Yang C, Jin P, Wang F (2022). Association between the triglyceride-glucose index and severity of coronary artery disease. Cardiovasc Diabetol.

[15] Lee EY, Yang HK, Lee J, Kang B, Yang Y, Lee SH, Ko SH, Ahn YB, Cha BY, Yoon KH (2016). Triglyceride glucose index, a marker of insulin resistance, is associated with coronary artery stenosis in asymptomatic subjects with type 2 diabetes. Lipids Health Dis.

[16] Song J, Xia X, Lu Y, Wan J, Chen H, Yin J (2022). Relationship among insulin therapy, insulin resistance, and severe coronary artery disease in type 2 diabetes mellitus. J Interv Cardiol.

[17] Escaned J, Collet C, Ryan N, De Maria GL, Walsh S, Sabate M, Davies J, Lesiak M, Moreno R, Cruz-Gonzalez I (2017). Clinical outcomes of state-of-the-art percutaneous coronary revascularization in patients with de novo three vessel disease: 1-year results of the SYNTAX II study. Eur Heart J.

[18] Takahashi K, Serruys PW, Fuster V, Farkouh ME, Spertus JA, Cohen DJ, Park SJ, Park DW, Ahn JM, Kappetein AP (2020). Redevelopment and validation of the SYNTAX score II to individualise decision making between percutaneous and surgical revascularisation in patients with complex coronary artery disease: secondary analysis of the multicentre randomised controlled SYNTAXES trial with external cohort validation. Lancet.

[19] Yan L, Li P, Wang Y, Han D, Li S, Jiang M, Cao X, Cao F (2021). The incremental prognostic value of the clinical residual SYNTAX score for patients with chronic renal insufficiency undergoing percutaneous coronary intervention. Front Cardiovasc Med.

[20] Park K, Ahn CW, Lee SB, Kang S, Nam JS, Lee BK, Kim JH, Park JS (2019). Elevated TyG index predicts progression of coronary artery calcification. Diabetes Care.

[21] Park GM, Cho YR, Won KB, Yang YJ, Park S, Ann SH, Kim YG, Park EJ, Kim SJ, Lee SG (2020). Triglyceride glucose index is a useful marker for predicting subclinical coronary artery disease in the absence of traditional risk factors. Lipids Health Dis.

[22] Li S, Guo B, Chen H, Shi Z, Li Y, Tian Q, Shi S (2019). The role of the triglyceride (triacylglycerol) glucose index in the development of cardiovascular events: a retrospective cohort analysis. Sci Rep.

[23] Gao S, Ma W, Huang S, Lin X, Yu M (2021). Impact of triglyceride-glucose index on long-term cardiovascular outcomes in patients with myocardial infarction with nonobstructive coronary arteries. Nutr Metab Cardiovasc Dis.

[24] Su WY, Chen SC, Huang YT, Huang JC, Wu PY, Hsu WH, Lee MY (2019). Comparison of the effects of fasting glucose, hemoglobin A(1c), and triglyceride-glucose index on cardiovascular events in type 2 diabetes mellitus. Nutrients.

[25] Akbar MR, Pranata R, Wibowo A, Irvan STA, Martha JW (2021). The association between triglyceride-glucose index and major adverse cardiovascular events in patients with acute coronary syndrome - dose-response meta-analysis. Nutr Metab Cardiovasc Dis..

[26] Zou S, Xu Y (2021). Association of the triglyceride-glucose index and major adverse cardiac and cerebrovascular events in female patients undergoing percutaneous coronary intervention with drug-eluting stents: a retrospective study. Diabetes Res Clin Pract.

[27] Neglia D, Aimo A, Lorenzoni V, Caselli C, Gimelli A (2021). Triglyceride-glucose index predicts outcome in patients with chronic coronary syndrome independently of other risk factors and myocardial ischaemia. Eur Heart J Open.

[28] Jin JL, Sun D, Cao YX, Guo YL, Wu NQ, Zhu CG, Gao Y, Dong QT, Zhang HW, Liu G (2018). Triglyceride glucose and haemoglobin glycation index for predicting outcomes in diabetes patients with new-onset, stable coronary artery disease: a nested case-control study. Ann Med.

[29] Pang S, Miao G, Zhou Y, Du Y, Rui Z, Zhao X (2022). Addition of TyG index to the GRACE score improves prediction of adverse cardiovascular outcomes in patients with non-ST-segment elevation acute coronary syndrome undergoing percutaneous coronary intervention: a retrospective study. Front Cardiovasc Med.

[30] Su J, Li Z, Huang M, Wang Y, Yang T, Ma M, Ni T, Pan G, Lai Z, Li C (2022). Triglyceride glucose index for the detection of the severity of coronary artery disease in different glucose metabolic states in patients with coronary heart disease: a RCSCD-TCM study in China. Cardiovasc Diabetol.

[31] DeFronzo RA, Inzucchi S, Abdul-Ghani M, Nissen SE (2019). Pioglitazone: the forgotten, cost-effective cardioprotective drug for type 2 diabetes. Diab Vasc Dis Res.

[32] Dormandy JA, Charbonnel B, Eckland DJ, Erdmann E, Massi-Benedetti M, Moules IK, Skene AM, Tan MH, Lefebvre PJ, Murray GD (2005). Secondary prevention of macrovascular events in patients with type 2 diabetes in the PROactive Study (PROspective pioglitAzone Clinical Trial In macroVascular Events): a randomised controlled trial. Lancet.

[33] Kernan WN, Viscoli CM, Furie KL, Young LH, Inzucchi SE, Gorman M, Guarino PD, Lovejoy AM, Peduzzi PN, Conwit R (2016). Pioglitazone after ischemic stroke or transient ischemic attack. N engl J med.

[34] Nissen SE, Nicholls SJ, Wolski K, Nesto R, Kupfer S, Perez A, Jure H, De Larochelliere R, Staniloae CS, Mavromatis K (2008). Comparison of pioglitazone vs glimepiride on progression of coronary atherosclerosis in patients with type 2 diabetes: the PERISCOPE randomized controlled trial. JAMA.

[35] Mazzone T, Meyer PM, Feinstein SB, Davidson MH, Kondos GT, D’Agostino RB, Perez A, Provost JC, Haffner SM (2006). Effect of pioglitazone compared with glimepiride on carotid intima-media thickness in type 2 diabetes: a randomized trial. Jama.

[36] Andrikou E, Tsioufis C, Andrikou I, Leontsinis I, Tousoulis D, Papanas N (2019). GLP-1 receptor agonists and cardiovascular outcome trials: an update. Hellenic J Cardiol.

[37] Akawi N, Checa A, Antonopoulos AS, Akoumianakis I, Daskalaki E, Kotanidis CP, Kondo H, Lee K, Yesilyurt D, Badi I (2021). Fat-Secreted ceramides regulate vascular redox state and influence outcomes in patients with cardiovascular disease. J Am Coll Cardiol.

