# Supplementary material for: Association of the triglyceride–glucose index with coronary artery disease complexity in patients with acute coronary syndrome
Source: Cardiovasc Diabetol. 2023 Mar 12;22:56. doi: 10.1186/s12933-023-01780-0 (PMC10010005; doi:10.1186/s12933-023-01780-0)
Supplement: Supplementary file 1 — Additional file 1: Table S1. The baseline characteristics based on tertiles of the baseline SYNTAX score. Table S2. Comparisons of the area under the ROC curves of the TyG index, FBG and TG. [file 12933_2023_1780_MOESM1_ESM.docx]

**Table S1 The baseline characteristics based on tertiles of the baseline SYNTAX score**

| **Variable** | **All subjects (n=1007)** | **bSS≤22 (n=815)** | **bSS＞22 (n=192)** | **P value** |
| --- | --- | --- | --- | --- |
| Age, years | 66.55 ± 11.41 | 65.73 ± 11.46 | 70.06 ± 10.53 | ＜0.001 |
| Female, n (%) | 284 (28.2) | 227 (27.9) | 57 (29.7) | 0.611 |
| BMI, kg/m2 | 24.31 ± 2.86 | 24.42 ± 2.84 | 23.84 ± 2.90 | 0.011 |
| Smoking, n (%) | 553 (54.9) | 455 (55.8) | 98 (51.0) | 0.230 |
| Previous PCI, n (%) | 84 (8.3) | 69 (8.5) | 15 (7.8) | 0.768 |
| COPD, n (%) | 55 (5.5) | 44 (5.4) | 11 (5.7) | 0.856 |
| Hypertension, n (%) | 655 (65.0) | 518 (63.6) | 137 (71.4) | 0.042 |
| Diabetes mellitus, n (%) | 360 (35.7) | 277 (34.0) | 83 (43.2) | 0.016 |
| AF, n (%) | 66 (6.6) | 48 (5.9) | 18 (9.4) | 0.079 |
| Previous Stroke, n (%) | 77 (7.6) | 57 (7.0) | 20 (10.4) | 0.108 |
| SBP, mmHg | 132.23 ± 21.43 | 132.52 ± 21.27 | 130.97 ± 22.07 | 0.368 |
| HR, bpm | 77.72 ± 14.78 | 77.25 ± 14.49 | 79.69 ± 15.86 | 0.040 |
| cTnT, pg/ml | 38.89 (12.03, 914.10) | 28.15 (11.14, 619.98) | 169.00 (21.36, 1877.75) | ＜0.001 |
| BNP, pg/ml | 112.80 (39.23, 315.53) | 94.60 (33.40, 261.40) | 213.80 (81.00, 539.70) | ＜0.001 |
| Scr, umol/L | 76.40 (64.80, 90.80) | 75.20 (63.70，88.60) | 82.50 (68.15, 103.35) | ＜0.001 |
| FBG, mmol/L | 6.95 ± 2.83 | 6.80 ± 2.70 | 7.58 ± 3.25 | 0.001 |
| TG, mmol/L | 1.60 ± 0.78 | 1.53 ± 0.66 | 1.90 ± 1.10 | ＜0.001 |
| CHO, mmol/L | 4.47 ± 1.22 | 4.44 ± 1.17 | 4.60 ± 1.42 | 0.113 |
| HDL, mmol/L | 1.15 ± 0.30 | 1.15 ± 0.29 | 1.16 ± 0.31 | 0.762 |
| LDL, mmol/L | 2.77 ± 0.90 | 2.75 ± 0.87 | 2.85 ± 1.01 | 0.173 |
| AMI, n (%) | 538(53.4) | 411(50.4) | 127(66.1) | ＜0.001 |
| **Diagnosis, n (%)** |  |  |  | ＜0.001 |
| UA | 469 (46.6) | 404 (49.6) | 65 (33.9) |  |
| NSTEMI | 229 (22.7) | 171 (21.0) | 58 (30.2) |  |
| STEMI | 309 (30.7) | 240 (29.4) | 69 (35.9) |  |
| **Angiographic data** |  |  |  |  |
| MVD, n (%) | 685 (68.0) | 501 (61.5) | 184 (95.8） | ＜0.001 |
| LM, n (%) | 54 (5.4) | 19 (2.3) | 35 (18.2） | ＜0.001 |
| Calcified lesions, n (%) | 138 (13.7) | 78 (9.6） | 60 (31.3) | ＜0.001 |
| Thrombosis, n (%) | 84 (8.3) | 60 (7.4) | 24 (12.5) | 0.021 |
| Long lesion, n (%) | 458 (45.5) | 350 (42.9) | 108 (56.3) | 0.001 |
| CTO, n (%) | 208 (20.7) | 112 (13.7) | 96 (50.0) | ＜0.001 |
| Number of stents | 1.46 ± 0.88 | 1.32 ± 0.75 | 2.05 ± 1.12 | ＜0.001 |
| Length of stents, mm | 38.34 ± 26.75 | 34.06 ± 22.77 | 56.49 ± 33.93 | ＜0.001 |
| bSS | 13.00 (8.00,20.00) | 11.00 (7.00, 16.00) | 27.50 (24.50, 31.23) | ＜0.001 |
| TyG index | 8.93 ± 0.57 | 8.87 ± 0.56 | 9.15 ± 0.59 | ＜0.001 |

The groups were stratified by the tertiles of the TyG index. BMI, body mass index; COPD, chronic obstructive pulmonary disease; AF, atrial fibrillation; SBP, systolic blood pressure; HR, heart rate; BNP, brain natriuretic peptide; Scr, serum creatinine; FBG, fasting blood glucose; TG, triglyceride; TC, total cholesterol; HDL-C, high density lipoprotein; LDL-C, low density lipoprotein; UA, unstable angina; STEMI, ST-segment elevation myocardial infarction; NSTEMI, non-ST-segment elevation myocardial infarction; MVD, multivessel disease; LM, left main disease; CTO, chronic total occlusion; bSS, baseline SYNTAX score; TyG index, the triglyceride–glucose index. Data are presented as mean ± SD, median (IQR) or n (%).

**Table S2 Comparisons of the area under the ROC curves of the TyG index, FBG and TG**

| **Variable** | Δ**AUC** | **95% CI** | **z** | **P value** |
| --- | --- | --- | --- | --- |
| TyG index vs. FBG | 0.0567 | 0.0138–0.0996 | 2.592 | 0.0095 |
| TyG index vs. TG | 0.0181 | -0.0137–0.0500 | 1.114 | 0.2651 |
| FBG vs. TG | 0.0386 | -0.0291–0.106 | 1.118 | 0.2638 |

ROC curve, ﻿receiver operating characteristic curve; TyG index, triglyceride–glucose index; FBG, fasting blood glucose; TG, triglyceride.
